# Influences of Maternal Care on Chicken Welfare

**DOI:** 10.3390/ani6010002

**Published:** 2016-01-05

**Authors:** Joanne Edgar, Suzanne Held, Charlotte Jones, Camille Troisi

**Affiliations:** 1 School of Veterinary Science, University of Bristol, Langford House, Langford BS40 5DU, UK; suzanne.held@bris.ac.uk (S.H.); j13429.2013@my.bristol.ac.uk (C.J.); 2 School of Biology, University of St Andrews, Queens Terrace, St Andrews, Fife KY16 9TS, UK; cat-5@st-andrews.ac.uk

**Keywords:** animal welfare, behaviour, chicken, domestic, hen, imprinting, laying, maternal, simulation, social learning

## Abstract

**Simple Summary:**

For a domestic chick, the mother hen is an important role model; chicks learn a great deal from their mother about what to peck, when to rest and how to behave when there is a threat. However, in large farms, natural brooding is not commercially viable and so chicks are hatched in large incubators and reared artificially. Chicks reared without a mother in this way are more fearful and more likely to develop behavioural problems, such as feather pecking. We discuss the important features of maternal care in chickens, the behavioural consequences of deprivation, and the welfare implications on commercial farms. We finish by suggesting ways to simulate natural maternal care to improve commercial chick rearing practice.

**Abstract:**

In domestic chickens, the provision of maternal care strongly influences the behavioural development of chicks. Mother hens play an important role in directing their chicks’ behaviour and are able to buffer their chicks’ response to stressors. Chicks imprint upon their mother, who is key in directing the chicks’ behaviour and in allowing them to develop food preferences. Chicks reared by a mother hen are less fearful and show higher levels of behavioural synchronisation than chicks reared artificially. In a commercial setting, more fearful chicks with unsynchronised behaviour are more likely to develop behavioural problems, such as feather pecking. As well as being an inherent welfare problem, fear can also lead to panic responses, smothering, and fractured bones. Despite the beneficial effects of brooding, it is not commercially viable to allow natural brooding on farms and so chicks are hatched in large incubators and reared artificially, without a mother hen. In this review we cover the literature demonstrating the important features of maternal care in domestic chickens, the behavioural consequences of deprivation and the welfare implications on commercial farms. We finish by suggesting ways to use research in natural maternal care to improve commercial chick rearing practice.

## 1. Introduction

As a precocial species, domestic chicks (*Gallus gallus domesticus*) are able to move around and feed independently shortly after hatching. These features have advantages for commercial egg and meat production, where chicks are hatched using artificial incubation and reared in large groups, without a mother hen. Although chicks are precocial, in a natural situation, maternal contact extends for 5–12 weeks [1]. During this period, the provision of maternal care strongly and beneficially influences the behavioural development of chicks. The artificial rearing of chickens may therefore lead to adverse and long-lived welfare consequences for the chicks, when we consider the important role of the mother hen. This review will cover the literature demonstrating the important features of maternal care in domestic chickens, the behavioural consequences of deprivation and the welfare implications on commercial farms. We will finish by suggesting ways to use research in natural maternal care to improve commercial chick rearing practice.

## 2. The Importance of Maternal Care

### 2.1. Imprinting

Maternal care in chickens is facilitated by filial imprinting, a process where newly hatched chicks learn to distinguish the shape and sound of their mother, and follow them [2]. This imprinting must occur within a sensitive period in the day or two after hatching. Following hatching, domestic chicks, and chicks of their wild ancestors—Red Junglefowl (*Gallus gallus*)—spend their early lives in close proximity to, if not under, their mother, especially in the first four days [1,3]. Hens maintain their brood as a discreet unit away from other individuals in the social group [1] at the periphery of the flock, with maternal behaviours persisting until the chicks are around 5–12 weeks of age [1,4,5]. The proximity that chicks maintain with the mother allows the expression of maternal care and the development of their social bond. This period of maternal contact which is facilitated by imprinting has important benefits for the correct development of sexual preferences, feeding behaviour, and behavioural synchrony. For instance, sexual imprinting is dependent on the parental phenotype of the opposite sex, with individuals seeking partners resembling similar characteristics [6]. This observation has been supported by avian cross-fostering studies showing that Great Tits (*Parus major*) reared by a Blue Tit (Cyanistes caeruleus) host failed in pairing with a conspecific mate [7]. Although sexual imprinting studies have not been experimentally applied to domestic chickens, evidence with other species provides grounds for suggesting that parental care, or even just the presence of adults of the opposite sex, has lifelong influences on future social interactions. That commercial domestic chicks have no access to adult birds during rearing may have implications for mate choice and productivity in flocks of breeder birds.

### 2.2. Communication between Mother and Chick

#### 2.2.1. Pre-Hatching Vocalisations

The mother and chicks begin to communicate as early as the day before hatching. When an embryo emits distress calls, the hen vocalises or moves onto the nest. Following this, the embryo becomes silent or begins to emit pleasure calls [8]. Vocalisations heard whilst still inside the egg are thought to help birds recognise their mother after hatching [9]. For domestic fowl, prenatal experience of maternal vocalisations is not necessary for the chicks to discriminate between individual hens, but it does reduce the age at which recognition is learnt post-hatching, allowing the chicks to recognise their own mother’s vocalisations by the time the hen and chicks leave the nest [10].

#### 2.2.2. Maternal Attraction and Alarm Calls

Following hatching, broody hens produce a variety of vocalisations directed towards their chicks; these can be characterised as either attraction or alarm calls [11]. Maternal attraction vocalisations—including roosting calls, maternal cluck calls, and feeding calls—are the primary way for the hen to attract her chicks and support the maintenance of the family unit [12]. The roosting call serves to attract the chicks to rest underneath the hen, usually just before night time. Roosting calls are characterised by long purring sounds which lack rhythm [11,13]. Cluck calls are slow, rhythmic clucks which attract and maintain the brood as a unit. Although hens emit these throughout the day to encourage following by the chicks, these increase as a response to chick stress [14,15]. The rhythmicity of cluck calls increases arousal and memory formation through a release of noradrenaline in the chick’s brain, which reinforces the learning of the desired behaviour [11,16]. The hen’s cluck calls enable chicks to differentiate between their mother and other hens [17,18]. Most chicks show this ability at four days of age—in time for leaving the nest—and this ability increases with age [10]. Early [10,19] and constant [17] exposure to this clucking stimulus is important to maintain chick responsive to the maternal call [19]. Studies have shown that the rate and intensity of maternal vocalisations also changes with the age of the chicks [10,20]. High rate and intensity of clucking by hens at a younger chick age allows the chicks to learn to recognise familiar from unfamiliar sounds [10]. Chicks are better able to discriminate their own mothers’ maternal vocalisations when visual stimuli are also present [17,21,22,23].

Alarm calls are clearly distinguishable from attraction calls by their higher volume and frequency [13]. Alarm calls include both predator and fear calls, which act to increase chick arousal in readiness for danger [11,24]. Unlike some attraction calls, neither type of alarm call has been shown to enhance memory formation in the chicks [11]. In fact, Gibbs & Summers [25] found that while moderate doses of noradrenalin facilitate memory formation in chicks during food or maternal calls, high doses of the same component inhibit memory consolidation during alarm calls. Field *et al.* [11] suggest that this memory inhibition could be an adaptive response which would cause the chicks to disregard what they are doing at a moment of danger, freeing their attention to focus on a potential threat.

#### 2.2.3. Maternal Feeding Display

When a broody hen discovers food, she will emit a characteristic high-pitched rapid vocalisation, which, along with pecking behaviour, attracts the chicks and encourages them to feed [4,26]. Very young chicks peck at food and non-food items indiscriminately; this initial pecking behaviour is not particularly sensitive to the consequences of ingestion and chicks learn little by conventional trial and error [27]. By eliciting the chicks’ pecking and attraction towards the place where the hen is pecking, the maternal feeding display facilitates the acquisition of adaptive foraging skills and knowledge of palatability by the chicks [27,28,29,30,31,32,33]. It has been suggested that local enhancement and social facilitation play an important role influencing the chicks’ behaviour towards food [34]. The role of the maternal feeding display in chick feeding behaviour is further supported by observational studies showing that young brooded chicks peck in the same locations as their mother [20,35]. Experimental evidence of a maternal influence comes from studies comparing feeding behaviour in brooded and non-brooded chicks. Wauters & Richard-Yris [34] found that, following a hen’s feeding display, the chicks expressed more feeding activity, mainly directed towards the same feeding source as the hen, and continued to feed even when the hen’s display finished.

Certain environmental and social variables are known to affect maternal food calling. For example, the intensity of food calling is positively correlated with the distance between the hen and chicks [36]. Environmental factors such as the presence of food [36,37], food quality [35,36,37], and quantity [36] all affect the maternal food display, including the intensity and length of food calls. Additionally, food calls are sometimes emitted in reply to chick distress calls [23,38]. Individual differences in feeding display also exist [37,39]; hens show strong food preferences [40], and maternal food calling reflects those preferences; more food calls are emitted in response to a preferred food source [37,40]. The motivational state of the hen also influences food call emissions; hungry hens emit more food calls than satiated hens [37].

Taken together, the food calls and pecking movements provide a combination of auditory and visual stimuli that increase arousal in the chicks [40], providing one of the contexts where social learning has an important role for chicks (for a review see [27]). However, unlike cluck calls, which immediately attract the chicks, food calls only attract the chicks when they have had prior experience of pecking and swallowing food [41], suggesting that the value and use of food calls depend upon the chicks’ experience. The sonic features of food calls are very helpful for the chicks to learn about the type and quality of food present [36,37]. Chicks peck at a higher and faster rate in response to higher call rate feeding displays [35,36,40]. Workman & Andrews [3] suggest that hens provide most of their information about food palatability before the chicks are eight days old. Within this period chicks react more rapidly to food calls as they grow older, further suggesting that learning might play a part in the chicks’ response [34].

In a commercial setting, to attempt to compensate for the indiscriminate pecking by non-brooded chicks, the chicks are provided with copious amounts of chick crumb, often on a paper surface to attract the chicks’ attention. Indeed, chicks are able to learn food preferences from same-age conspecifics; observational research on a semi-domesticated population of Red Junglefowl has highlighted juvenile copying as a learning method for food acquisition [4]. However, the lack of an experienced mothers’ direction may have implications for the development of pecking preferences, with chicks directing pecks towards inappropriate areas such as the feathers of conspecifics [42,43] (see Section 3).

### 2.4. Teaching

We have so far concentrated on the information that chicks are gaining from an essentially passive mother hen. However, early work suggested that the maternal feeding display is affected by the chicks’ behaviour. In 1971, Stokes [4] found that hens cease food calling when the chicks approach and feed. Further work showed that hens emit food calls especially when their chicks are not feeding or have been at some distance for several seconds [37,40]. Whether the hen is able to utilise information from the chicks to facilitate learning has been the subject of one study, the results of which point towards potential teaching behaviour in the domestic fowl. According to Caro & Hauser’s [44] definition, for a behaviour to be classified as teaching there are three criteria to fulfil. First, the demonstrator must modify its behaviour only in the presence of the naïve observer. Second, this modified behaviour must incur a cost to the demonstrator, or at least no direct benefit. Finally, as a result of the modified behaviour, the naïve observer must acquire knowledge or skills that it would not have learned otherwise, or would not have learned as rapidly. Nicol & Pope [45] provided hens with the choice between two coloured food sources: one palatable, the other unpalatable (quinine was added). Each hen’s brood of chicks were split into two notional groups: one group were trained to feed from the same colour as the hen, and the other group were trained to feed from the opposite colour. Subsequently, when the hen watched chicks from the second group—those that were pecking at the coloured food source that was seemingly unpalatable to the hen—the hen increased food pecking, ground pecking, and scratching. Using Caro & Hauser’s criteria, we can see strong evidence for the first criterion—modification of the demonstrator’s behaviour only in the presence of the observer—but weaker ones for the other two. Regarding the second criterion, one could suggest that there is a cost to this behaviour or at least no immediate benefit, as the feeding display increases the hen’s latency to feed and increases competition for food [33]. Relating to the third criterion of Caro & Hauser’s definition, it could be claimed that there might be an increase in chick foraging skills due to the improved opportunity to identify palatable from unpalatable food. This claim is indirectly supported by the transmission of arbitrary maternal food preferences from hens to offspring [33,40]. Further studies are needed to assess whether the chicks modify their feeding behaviour in response to the hen’s display. However, in Nicol & Pope’s experiment, the hen’s change in behaviour was not as a reaction to chick disgust display (e.g. beak wiping and head shaking), but rather as a combined assessment of the hen’s prior knowledge of food palatability and the chicks feeding choice. The study showed that hens are sensitive to errors made by the chicks, and confirms the important influence of maternal care on chick behavioural development.

### 2.5. Behavioural Synchrony

Maternal influences have long been known to facilitate the modulation of ultradian rhythms in mammalian young [46,47]. In precocial birds, ultradian rhythms are defined in terms of active and inactive periods [48]. Synchronisation of ultradian rhythms allows individuals within a group to stay together [49], and helps to group individuals according to their different motivations [50]. In precocial birds, being behaviourally synchronised in this way helps to provide a means of thermoregulation [51]. Very young chicks are unable to regulate their body temperature and so, in a natural situation, chicks spend a large proportion of time resting under and gaining warmth from their mother, in relative darkness. One study found that day-old brooded chicks spent 60% of their time resting under the hen. This decreased sharply during the first two weeks, was stable at around 10% from 13 days of age, and was scant at 25 days of age, in line with the growth of adequate feather cover to thermoregulate [52]. Although non-brooded chicks show ultradian rhythms for the first three days [53], this synchrony disappears without a mother hen. Natural brooding results in behavioural and diurnal synchronisation within the brood, where active and inactive behaviours are performed at the same time by all brood members [54]. Riber *et al.* [54] found no difference between total time spent active between the brooded and non-brooded chick groups, but the length of activity bouts and synchronisation were significantly greater in brooded chicks. Wauters *et al.* [55] compared activity levels in brooded and non-brooded chicks and found that whilst brooded and non-brooded chicks engaged in the same behaviours for a similar amount of time, the activity bouts were much longer in brooded chicks. Brooded chicks were more behaviourally synchronised and also demonstrated a greater use of the available space. Mother hens also function as moving heaters, positioning themselves near to resources such as food and water, where they encourage chicks to feed [56], further synchronising the brood. In contrast, in a commercial setting, chicks experience a continuous period of light and dark and are provided with static or whole house heating, meaning that active and inactive chicks are not separated. The welfare implications that may arise from the continuous periods of light and dark on commercial farms is discussed in Section 3.2.

### 2.6. Mediating the Chicks’ Fear and Stress Response

Hens respond behaviourally and physiological to exposure of their chicks to a stressor [14,57]. In response to a short-term stressor (air puff) applied to the chicks hens showed an increased heart rate, time spent alert and cluck calling, and a decreased eye temperature and time spent preening. Further work suggested that hens’ response to chick stress depends on their cognitive appraisal of the situation, but that they also react to stress cues from their chicks [15]. Indeed the mother hen has an important role in mediating the chicks’ response to threats; the presence of the chicks’ mother acts to buffer the stress response of domestic chicks during the application of an air puff [57]. Chicks showed a return towards baseline levels of preening and ground pecking immediately after this stressor when their mother was present compared to absent.

Similarly, brooding is known to have a general buffering effect on chick fearfulness. In response to a human standing up, non-brooded chicks showed greater frequency of flight responses, more time standing, and increased perch use, when compared to brooded chicks [58]. In open field tests, four-week old non-brooded chicks spent less time walking and more time freezing and vocalising [52]. Five six-week old brooded chicks were more active in the open-field test, indicating that they are less fearful [59]. The fear-alleviating effect of brooding is further supported by Campo *et al.*’s [56] study which found that six-week old non-brooded chicks had longer durations in tonic immobility than their brooded counterparts. Evidence of physiological responses to brooding have come from a study showing that non-brooded chicks had a lower whole-blood 5-HT (serotonin) concentration than birds from the other treatments, characteristics associated with high feather pecking lines of chicks [60].

However, contradictory evidence of the effects of brooding on fear have been found. There was no significant difference in tonic immobility when brooded and non-brooded two to three-week old chicks were placed in a novel situation [61]. One possible reason for the conflicting finding is that chicks’ response to threats mirrors that of their mother; with low responding mothers transmitting their low arousal to their chicks. Indeed this is in accordance with work showing that mothers that showed lower arousal levels—indicated by a lower heart rate increase in response to chick stress—were more effective social buffers for their chicks [57]. Mothers thus shape their chicks’ stress response according to their own assessment of a potential threat. Indeed, cross fostering studies using Japanese quail (*Coturnix japonica*) also point to individual differences in maternal responsiveness to threats which are passed on to chicks. Bertin and Richard-Yris [62] found that quail chicks of mothers that were not habituated to humans were more fearful than the chicks of mothers that were habituated, suggesting maternal transmission of fear towards humans. Houdelier *et al.* [63] cross fostered quail chicks, selected for either higher (LTI) or lower fearfulness (STI) and from a control line (C). The chicks were fostered by LTI or STI mothers. They found that, whatever their genotype, the fearfulness of chicks fostered by LTI mothers was higher than that of chicks fostered by STI mothers. However, the least fearful chicks (STI) were the least affected by early experience with mothers suggesting that genetic background affected the strength of the maternal effects. The overall strong maternal influence on chick stress and fear may have implications on commercial farms, where non-brooded chicks may not learn how to respond appropriately to ambiguous stimuli. This may result in an over-reaction to non-threatening stimuli, such as panic responses caused by humans entering a shed, or on the flip side, a reduced vigilance to predation risk.

## 3. Animal Welfare Implications and Maternal Simulation

Despite the beneficial effects of maternal care, it is not commercially viable to allow brooding on farms. Studies have demonstrated that brooded chicks show a reduced feed conversion [40], decreasing growth rate [64]. Indeed, broodiness itself has a detrimental effect on production; broody hens cease laying and aggressively defend the nest area, taking up space that could be utilised by hens in lay. Consequently, maternal behaviour has been increasingly selected out of commercial laying hen strains, to the point where broody hens are rare amongst commercial breeds.

However, we have described the strong effects that a mother hen has on chick behaviour through mechanisms of imprinting, vocalisations, social learning, and behavioural synchrony. Studies comparing brooded and non-brooded chicks have found that brooded chicks are more active—performing more floor pecking and dust bathing—than non-brooded chicks [43,52]. Brooded chicks also perform more sustained feeding behaviours [40], are more reactive to vocalisations [52], less fearful [52,61], less aggressive [65], and have a higher motivation for social contact [61] than non-brooded chicks. These behavioural effects are likely to have consequences within a commercial environment, where chicks are reared in large groups, without a mother. For example, feather pecking—when birds peck at and remove feathers from conspecifics—is a serious welfare and economic problem [66,67]. It has been hypothesised that the presence of a mother hen early in life could prevent the onset of feather pecking by encouraging the chicks to direct their pecks towards more appropriate stimuli, such as the ground or litter [42,43,68]. Indeed, studies have found that feather pecking is negatively correlated with ground pecking [69,70,71]. By synchronising the chicks’ behaviour, resting conspecifics are kept apart (spatially and temporally) from active conspecifics that might direct feather pecks toward them. This maternal protection against feather pecking might not materialise until the chicks are older; whilst Roden & Wechsler [58] found similar levels of feather pecking in groups of brooded and non-brooded one-week old chicks, Riber *et al.* [43] showed that feather pecking and mortality were higher in non-brooded chicks at 20 and 24 weeks of age. Another hypothesised mechanism for reduced feather pecking in brooded chicks is through the fear-mediating effect of brooding (discussed in Section 2.6). More fearful chicks show an increased inclination to feather peck as adults [72] and low pecking genetic lines of chicken show reduced fear responses in open field tests [71]. Aside from the fact that fear is itself a negative subjective state and therefore adversely affects welfare, increased fear can cause panic responses which, in large groups, is hypothesised to lead to smothering and fractured bones [73]. Additionally, fear is associated with reduced ranging [74], although the causality has not yet been determined.

Although the mechanisms of the behavioural changes associated with being brooded are not yet understood, it is possible to simulate a number of features of maternal care, including vocalisations, as well as inherent properties of mother hens comprising vocalisations, warmth and darkness, as well as olfaction.

### 3.1. Vocalisations

Chick behaviour is heavily influenced by the mother’s vocalisations [16,17,75]. Earlier work identified maternal cluck calls as a key component of the maternal response to chick stress [14,15] and pointed to their potential use during times of stress. Subsequently, Edgar *et al.* [76] found that playback of maternal cluck calls decreased stress response in non-brooded chicks, as measured using stress induced hyperthermia, but only when playback was at a lower duration—simulating a lower responding mother. The chicks responded regardless of whether they had prior experience of the playback, suggesting that maternal cluck calls might be a primary reinforcer for domestic chicks. Crucially, however, vocalisation playback did not change the chicks’ behavioural (freezing) response to the stressor, indicating that additional features of maternal care are required to further reduce stress. Although simulation of this particular call type proved only partially useful, there is potential for its use alongside other maternal features. Indeed playback of other vocalisations may prove useful. For example as discussed in Section 2.2, roosting and feeding calls play important roles in encouraging the chicks to rest and to direct their pecking [4,11,13,26,40]. Despite the fact, noted in Section 3, that a mother hen actually reduces feed conversion [40], promising small scale experiments showed that playing recordings of maternal food calls near to the feeder improved feed conversion and increased chick weight [77]. Surprisingly, the effects of playback of these calls on chick behaviour and welfare have not yet been studied but remain a potentially promising maternal simulation.

### 3.2. Warmth and Darkness

**Figure 1:**
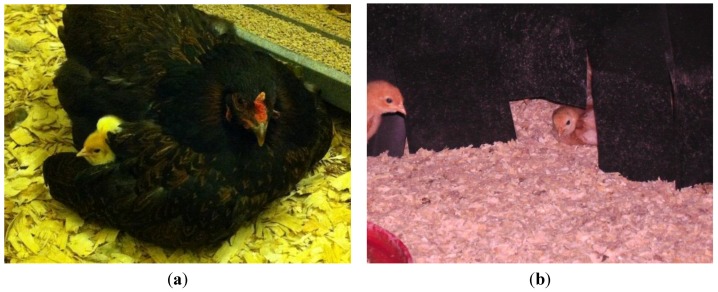
(**a**) Left: Mother hens provide an important source of warmth and darkness for their chicks (Photo credit—J Edgar); (**b**) Right: Close up of the plastic fringed entrance to a dark brooder. Dark brooders have been employed in a commercial setting to simulate the warmth and darkness of a broody hen (Photo credit—Anne-Marie Gilani).

In the first few weeks of life, chicks are unable to thermoregulate and so they naturally spend a large proportion of time resting under and gaining warmth from their mother, in relative darkness (see Figure 1a). In contrast, in the commercial setting, chicks are provided with artificial radiant heat from static brooders or whole house heating, and experience continuous light periods. In this situation, behaviours become unsynchronised and chicks may disturb and direct feather pecks towards resting conspecifics [78]. Chicks on a light cycle which mimicked natural brooding (40 min light: 40 min dark periods throughout the main light period) rested more than control chicks with a continuous period of light and then dark. The treatment chicks had highly patterned levels of activity without compromised weight or feed efficiency [79]. Dark brooders—devices from which heat is provided under the canopy of dark plastic fringes to block out the light—simulate both the darkness and the warmth of a broody hen (see Figure 1b). Dark brooders help to reduce feather pecking in later life [80,81] and have no detrimental effects on production [78]. Dark brooding is an example where consideration of natural maternal behaviour can lead to the application of practical on-farm solutions to welfare problems and is the first and only commercial application of a maternal simulation.

### 3.3. Olfaction

Although brooded chicks generally show lower fear responses than those reared artificially [59,61,82], the mechanism by which the mother hen reduces chick fear response is currently poorly understood. Evidence of an inherent maternal calming effect comes from studies of broody hen preen gland secretions, proposed to be analogous to appeasing pheromones in mammals. MHUSA (Mother Hens’ Uropygial Secretion Analogue), a synthetic analogue of this secretion (composed of 12- to 18-carbon fatty acid methyl esters) has been isolated and tested for its effects on commercially-housed broiler chicks. At the end of the rearing period, MHUSA treated broiler chicks were heavier, showed less scarring, and reduced physiological indicators of stress at slaughter [83].

These results show that MHUSA has an effect on modifying chick behaviour and fear, but its effects on behavior and consequences for welfare in commercially housed chickens has not yet been determined.

## 4. Welfare Consequences Caused by a Lack of Opportunity for Maternal Behaviour

As discussed, there are likely potential welfare implications for the chicks caused by the absence of a mother-offspring bond. For *the mother hen*, any welfare consequences need to be considered in terms of a lack of opportunity to display a natural behaviour. Indeed, animal welfare is often defined in terms of the ethical concept of “naturalness” [84], a concept which can drive consumer product choice [85]. However, it could be argued that commercial hens that rarely go broody would not suffer from a lack of opportunity to display maternal behaviour because the motivation to express maternal behaviour is being selected out of commercial breeds. A natural mother/offspring bond is considered important in dairy farming, where the length of time a calf remains with its mother can vary, and is considered to have differing welfare implications for both the cow and her calf (for a review see [86]). In the poultry industry, consisting of many more individual animals with relatively shorter lifespans, the damage to productivity caused by a natural mother-offspring bond might generally be considered too great. For those hens that do go broody, the extent to which hens’ welfare is affected by the absence of a mother-offspring bond needs to be weighed up in terms of the lack of opportunity for hens to experience positive aspects of maternal behaviour.

## 5. Conclusions

We have discussed how the mother hen influences the behavioural development of her offspring. As a precocial species, domestic chicks are self-sufficient after hatching, meaning that maternal behaviour has become redundant on farms. For commercial egg and meat production, chicks are hatched using artificial incubation and reared in large groups, without a mother hen. Maternal behaviour, from laying through to chicks’ independence, has a substantial influence on chick development. As well as serving an important protective and heat-providing function, the mother hen attracts chicks to profitable food items, and also redirects their attention away from harmful or non-profitable items [45]. This is especially important during the first few days of life, when pecking behaviour is not particularly sensitive to the consequences of ingestion, and chicks learn little by conventional trial and error [27]. In a commercial setting, the lack of opportunity to learn about species-specific appropriate behaviour may have implications for the ability to display normal behaviour later in life. Since maternal care has detrimental effects on some production parameters, research should focus on determining the important features of maternal care that could be artificially simulated to improve welfare and can be practically integrated into commercial chick rearing practice.
